# Adult attention-deficit/hyperactivity disorder is associated with reduced norepinephrine transporter availability in right attention networks: a (S,S)-O-[^11^C]methylreboxetine positron emission tomography study

**DOI:** 10.1038/s41398-019-0619-y

**Published:** 2019-11-15

**Authors:** Christine Ulke, Michael Rullmann, Jue Huang, Julia Luthardt, Georg-Alexander Becker, Marianne Patt, Philipp M. Meyer, Solveig Tiepolt, Swen Hesse, Osama Sabri, Maria Strauß

**Affiliations:** 1 Department of Psychiatry and Psychotherapy, University of Leipzig Medical Center, 04103 Leipzig, Germany; 2 Department of Nuclear Medicine, University of Leipzig Medical Center, 04103 Leipzig, Germany

**Keywords:** ADHD, Physiology

## Abstract

The norepinephrine transporter (NET) has been suggested to play a critical role in attention-deficit/hyperactivity disorder (ADHD). In this prospective controlled study we tested the a-priori-hypothesis that central NET availability is altered in adult ADHD patients compared to healthy controls. Study participants underwent single positron emission tomography-magnetic resonance imaging (PET-MRI). MRI sequences included high resolution T1-MPRAGE data for regions of interest (ROI) delineation and voxel-based morphometry (VBM) and T2-weighted fluid-attenuated inversion-recovery for detection and exclusion of pathological abnormalities. NET availability was assessed by NET-selective (S,S)-O-[^11^C]methylreboxetine; regional distribution volume ratios (DVR) were calculated based on individual PET-MRI data co-registration and a multi-linear reference tissue model with two constraints (MRTM2; reference region: occipital cortex). VBM analysis revealed no difference in local distribution of gray matter between the 20 ADHD patients (9 females, age 31.8 ± 7.9 years, 488 ± 8 MBq injected activity) and the 20 age-matched and sex-matched control participants (9 females, age 32.3 ± 7.9 years, 472 ± 72 MBq). In mixed-model repeated-measures analysis with NET availability as dependent and ROI as repeated measure we found a significant main effect group in fronto-parietal-thalamic-cerebellar regions (regions on the right: *F*_1,25_ = 12.30, *p* = .002; regions on the left: *F*_1,41_ = 6.80, *p* = .013) indicating a reduced NET availability in ADHD patients. None of the other investigated brain regions yielded significant differences in NET availability between groups after applying a Benjamini-Hochberg correction at a significance level of 0.05. Overall our findings demonstrate the pathophysiological involvement of NET availability in adult ADHD.

## Introduction

Adult attention-deficit/hyperactivity disorder (ADHD) is an underrecognized chronic disorder with childhood-onset characterized by attention deficit, impulsivity and hyperactivity. The worldwide prevalence of ADHD is estimated to be 2.8%^1^, in Germany as high as 4.7%^2^. Moreover, a mortality rate of 5.8% has been found in ADHD, highest in individuals diagnosed in adulthood and mainly driven by deaths from unnatural causes, e.g., accidents^3^. ADHD is often accompanied by other comorbidities, such as depression, anxiety and conduct disorder^4^. ADHD symptoms have a detrimental impact on social, financial and professional functioning^5^—the risk of losing one’s job for example is three times higher in ADHD. Not surprisingly, the economic impact of adult ADHD places a significant burden on society. The gold standard treatment is pharmacotherapy, but 30% of adult patients with ADHD do not respond to medication^6,7^.

Patients with ADHD show executive functioning deficits, impaired attention and behavioral control, relating to brain areas modulated by noradrenergic transmission^8,9^ and norepinephrine transporter (NET) expression. Animal models of ADHD demonstrate the involvement of the central locus-coeruleus norepinephrine (LC-NE) system in behavioral control and the attentive process^10,11^. Drugs modulating NE transmission and targeting NET, such as atomoxetine or methylphenidate, are effective in ADHD patients^6^ and have been shown to significantly occupy NET in vivo at clinically relevant doses^12,13^. There is ample and clear evidence that the LC-NE system is involved in regulating wakefulness and arousal^14,15^ shown to be reduced in patients with adult ADHD^16–18^.

While NE is released throughout the entire mammalian brain, evidence from structural and functional neuroimaging studies suggests that there are right hemispheric deficits in ADHD, specifically reduced overall cerebral volume including fronto-parietal areas, basal nuclei, globus pallidus and cerebellum^19^. Moreover, studies show functional abnormalities in two distinct domain-dissociated networks: (1) the fronto-parietal-thalamic-cerebellar network for attention and (2) the fronto-basal-ganglia network for inhibition^20–22^. The normalizing effect of methylphenidate and atomoxetine on the right fronto-parietal-thalamic activation during sustained attention^23^, known to be challenging for patients with ADHD^24^, corroborates the validity of the first model, further supported by the benefits of stimulation of the right dorsolateral frontal cortex on symptoms of inattention in patients with adult ADHD^25^. Concerning the second model, evidence from lesion and imaging studies suggest a direct pathway from the right inferior frontal cortex to the basal ganglia^26,27^ in stopping tasks. Importantly, dysfunctions of the prefrontal-striatal systems are implied in ADHD^28–31^ and basal ganglia are areas of NET expression, albeit less dense than in the LC^32,33^.

So far, changes in NET availability have not been described in adults with ADHD. One study exploring NET binding potential using radioligand (S,S)-[^18^F]FMeNER-D_2_^33^ in selected regions of interest (ROI) did not yield any significant differences between adults ADHD patients and healthy controls. However, using NET-selective (S,S)-O-[^11^C]methylreboxetine, a previous study found associations between central NET availability and trait impulsivity in twenty healthy individuals^34^.

In the present study we tested the hypothesis that central NET availability is altered in adult patients with ADHD compared to healthy control subjects. We further explored whether NET availability is associated with ADHD symptom severity, neuropsychological and neurophysiological measures. To evaluate differences in NET availability, PET and NET-selective (S,S)-O-[^11^C]methylreboxetine (MRB) was applied in unmedicated adult patients with ADHD and age-matched and sex-matched healthy controls. We applied a ROI approach, focusing on areas known to be functionally relevant for ADHD that are linked to the LC-NE system.

## Materials/subjects and methods

### Study participants

The study was conducted in accordance with the ICH Guideline for Good Clinical Practice (GCP) and the declaration of Helsinki, and was approved by the local ethics committee (EC number 155/15-ff) and the German *Bundesamt für Strahlenschutz*/Federal Office for Radiation Protection (number Z 5–22461 2-2016-008). Between February and December 2018, outpatients (age ≥ 18) meeting DSM-V criteria for ADHD based on mental status examination, semi-structured Diagnostic Interview for ADHD in adults (DIVA 2.0^35^, adult ADHD Self-Report Scale Symptom Checklist (ASRS 1.1^36^), German version of the Wender-Utah-Rating-Scale (WURS-k^37^ ≥ 30; or known ADHD diagnosis in childhood) were consecutively recruited from the Adult ADHD outpatient center of the Department of Psychiatry and Psychotherapy of Leipzig University. Age- and sex-matched healthy control participants were recruited from the local community through advertisements. Patients had to be free of any psychotropic medication (ADHD-specific medication, antidepressants, sedatives, z-hypnotics, neuroleptics) and drug use for at least one month prior to the first screening visit; controls had to be naïve to psychotropic medication and had to be free of any drug use for at least one month prior to the first screening visit. Healthy controls received financial reimbursement.

Written informed consent was obtained from all participants at the beginning of the first screening visit after ensuring that all participants were able to understand the study protocol. To evaluate the general health of all participants, physical and neurologic status and routine laboratory tests were performed. Patients with serious psychiatric (acute severe depressive episode, suicidality, psychotic symptoms, bipolar disorder, schizophrenia, psychosis), neurological (history of brain surgery, significant brain malformation or neoplasm, head injury, stroke, epilepsy, neurodegenerative disorder), and/or severe somatic comorbidities were excluded. Any contraindication to MRI (e.g., heart or brain pacemaker, metal implants in the head/neck) and pregnancy also lead to patients’ exclusion. A multi-drug urine test was performed at the first screening. Women underwent a urine pregnancy test before the PET-MRI measurement.

### Image acquisition, image data processing, and quantification

PET-MRI data were acquired on a hybrid PET-MRI device (Biograph mMR, Siemens, Erlangen) and processed as previously described^38^. MRI sequences included high resolution T1-MPRAGE data for ROI delineation, and voxel-based morphometry (VBM) and T2-weighted fluid-attenuated inversion-recovery (FLAIR) for detection and exclusion of pathological abnormalities. Dynamic PET imaging was performed after intravenous bolus injection (90 s) of 480 ± 51 MBq [^11^C]MRB for 90 min (23 frames: 4 × 15 s, 4 × 60 s, 5 × 120 s, 5 × 300 s, 5 × 600 s). For attenuation correction we applied the MR-based μ-map estimation by Catana et al.^39^. Using PMOD (version 3.5, PMOD Technologies, Zurich, Switzerland), PET data motion correction was performed, as well as the generation of the regional DVR maps. Accordingly, we applied the multi-linear reference tissue model with 2 constraints (MRTM2^40^) using a fixed clearance rate of k2’ = 0.0238 min^−1^ and the occipital cortex serving as the reference region. We used PMOD’s Neuro module for automatic individual ROI delineation of the merged Automated Anatomical Labeling (AAL^41^) atlas. For the Harvard Ascending Arousal Network (AAN^42^) probability atlas we calculated the inverse transformation matrix of the spatial normalization to the MNI-space based on the T1-MPRAGE in SPM12 (Wellcome Centre for Human Neuroimaging University College London https://www.fil.ion.ucl.ac.uk/spm/software/spm12/) and applied the inverse matrix to the AAN atlas to transfer the atlas to the participant’s individual space.

For VBM analysis we used the CAT12 (http://www.neuro.uni-jena.de/cat/) running in SPM12. In short, individual T1 data were spatially normalized, segmented in gray matter, white matter and cerebrospinal fluid and smoothed using a Gaussian filter with 8 mm full-width half-maximum filter size.

### Questionnaires

The DSM-IV subscales of the Conners’ Adult ADHD Rating Scale Self-Report (CAARS-SR–long version^43,44^) were applied at the screening visit to assess ADHD symptom severity. The Structured Clinical Interview for DSM-IV Axis I and Axis II disorders^45,46^ was performed to exclude comorbid psychiatric disorders. IQ was determined with vocabulary-based IQ screening (*Wortschatztest*, WST^47^). Substance use was evaluated with Alcohol Use Disorders Identification Test^48^ and Drug Use Disorders Identification Test^49^. The Beck Depression Inventory-II^50^ (BDI) was applied to assess depressive symptom severity.

### Neuropsychological measures

The sustained attention subtest of the computerized test of attentional performance (TAP^51^) was used to assess attention and behavioral control at the screening visit. In this 15-min test (3 × 5-min blocks), a sequence of stimuli ranging in color, shape, size and filling, is presented on the monitor. A target stimulus occurs whenever it corresponds in shape with the preceding stimulus (e.g., the same shape but with different color, size and filling). Participants were instructed to press a button as quickly as possible when the target stimulus appeared. The probability for the appearance of the target-stimuli was 12 percent (450 trials, 54 targets, stimulus onset asynchrony: 2 s). Errors of omission were defined as number of targets that were presented but not responded to and were used as measure of sustained attention. Errors of commissions were defined as number of non-targets that were responded to and used as measure of behavioral control (inhibition/impulsivity).

### Neurophysiological EEG measures

We utilized standardized low-resolution brain electromagnetic tomography (sLORETA) to estimate the prefrontal current density of the theta band (6.5–8.0 Hz). To calculate the current source density of prefrontal EEG theta we recorded EEG signals at the screening visit with Ag/AgCl electrodes using a QuickAmp amplifier (Brain Product GmbH, Gilching, Germany) from 31 electrode positions according to 10–20 system under a 15-min resting condition. Impedance of each electrode was kept below 10kΩ. The raw EEG was firstly down sampled to 256 Hz and then a band pass filtering between 0.1 and 100 Hz was performed. For all participants, the first 5 minutes of entire EEG was segmented and imported into sLORETA, to analyze the cortical three-dimensional distribution of scalp current density^52^. Each EEG was processed via a sLORETA cross-spectrum analysis; the software then calculated, based on the cross-spectrum data for the theta band (6.5–8.0 Hz), the current source values for each voxel. The current density values in the right and left dorsolateral prefrontal cortices (indexed by Brodmann Area 9; BA9) were averaged over all voxels within this ROI.

### Statistical analysis

To investigate whether there are significant differences between ADHD patients and healthy controls regarding demographic variables and clinical variables Chi-square tests, t-tests for independent sample comparison, or Mann–Whitney *U* tests (dependent on scale of measurement and normality of data) were performed to identify relevant covariates. To determine whether the NET availability is different between ADHD patients and healthy controls mixed-model repeated-measures (MMRM) analyses were applied with NET availability as outcome and ROI as repeated factor, and an unstructured covariance matrix. Models were implemented with restricted maximum likelihood algorithm. Fixed class effects of group (patients versus controls), ROIs, the interaction of group and ROI, BDI score as covariate, as well as random effects for subjects, matched participants and intercept were included. Separate models for attention—(including superior frontal gyrus, precuneus, angular and supramarginal gyri, cerebellum with crus and thalamus) and behavioral control-related (including inferior frontal gyrus, anterior cingulate, supplementary motor area, nucleus caudate, putamen, pallidum) ROIs based on the AAL probability atlas were calculated for the right and left side, as well as a model for LC and Raphe Nuclei based on the AAN probability atlas. The statistical significance of the main effect group or the interaction group x ROI was utilized to determine whether the NET availabilities in selected ROIs are different across the groups. In cases of significant main effects or interactions, Spearman correlation analyses between the mean DVR of respective ROIs and clinical (T-scores of CAARS DSM-IV subscales inattention and hyperactivity/impulsivity), neuropsychological (T-scores of numbers of omission and commission errors during the 15-min sustained attention test) and neurophysiological measures (EEG theta current source density of BA9) were conducted; in case of significant findings, group was included as covariate to estimate the effect of group. All statistical analyses were computed using SPSS, version 24.0 for Windows (IBM Corp., Armonk, NY, USA). The two-tailed significance level was set at *p* = .05. The corrected significance level for multiple tests according to Benjamini and Hochberg^53^ was *p* = .014.

We used VBM to investigate differences in local distribution of gray matter between ADHD patients and age-matched and sex-matched healthy control participants. The modulated and spatially normalized gray matter images were fed into SPM12, where we applied a two-sample *t*-test with total intracranial volume as a confound to correct for different brain sizes. All clusters with a combined threshold of *p* < .001 uncorrected at peak level and *p* < .05 family-wise error corrected at cluster level were considered as significantly different.

## Results

### Description of sample

The final study sample consisted of 20 adult patients with ADHD (11 males, age 31.8 ± 7.9 years, 488 ± 8 MBq injected activity) and 20 age- and sex-matched healthy controls (11 males, age 32.3 ± 7.9 years, 472 ± 72 MBq). Sample characteristics are presented in Table 1. Groups did not differ in basic demographic and biological characteristics (all: *p* > .10), however, a significant between-group difference in BDI sum scores was found (*p* < .0001). Thus, we decided to integrate BDI sum score in multivariate analysis.

**Table 1:** Demographic and clinical characteristics

	ADHD (*n* = 20)	Control (*n* = 20)	All (*n* = 40)	*p*-value
Characteristic				
Number of males	11 (55%)	11 (55%)	22 (55%)	1.000
Age (years)	31.8 (7.9) 22–49	32.3 (7.9) 20–52	32.1 (7.8) 20–52	.843
Weight (kg)	80.4 (14.0) 49–107.5	79.4 (11.16) 58–98	79.9 (12.5)	.373
Height (cm)	174.8 (8.1) 156–187	175.3 (10.3) 156–196	175.1 (0.9)	.213
Smoking status, yes/no	7/13 (35/65%)	6/14 (30/70%)	13/27 (32.5/67.5%)	.736
BMI (kg/m^2^)	26.2 (3.7) 19.5–32.7	25.7 (2.0) 21.9–28.9	26.0 (3.0)	.610
Handedness				.198
Right handed	17 (85%)	20 (100%)	37 (92.5%)	
Left handed	2 (10%)	0 (0%)	2 (5%)	
Ambidextrous	1 (5%)	0 (0%)	1 (2.5%)	
Beck depression inventory, sum score	9.9 (11.3) 0–40	1.4 (2.5) 0–9	5.7 (9.2)	<.0001
WST_IQ	107.3 (9.4) 95–133	106.9 (8.1) 92–122	107.1 (8.7)	.889
WURS-K, sum score	42.3 (9.5) 25–60	9.6 (8.5) 0–24	25.9 (18.8)	<.0001
CAARS DSM-IV Inattention, *T*-score	84.4 (6.7) 70–90	44.5 (8.6) 36–66	64.4 (21.6)	<.0001
CAARS DSM-IV hyperactivity/ impulsivity, *T*-score	74.8 (12.1) 54–90	43.1 (5.4) 35–54	58.9 (18.5)	<.0001

Annotations: Entries are means (±standard deviations) or numbers (%) and range

*BMI* body mass index, *WST* Wortschatztest; *WURS-k* Wender Utah rating scale, *CAARS* Conners’ Adult ADHD rating scales

### Description of distribution volume ratios DVRs

**Fig. 1:**
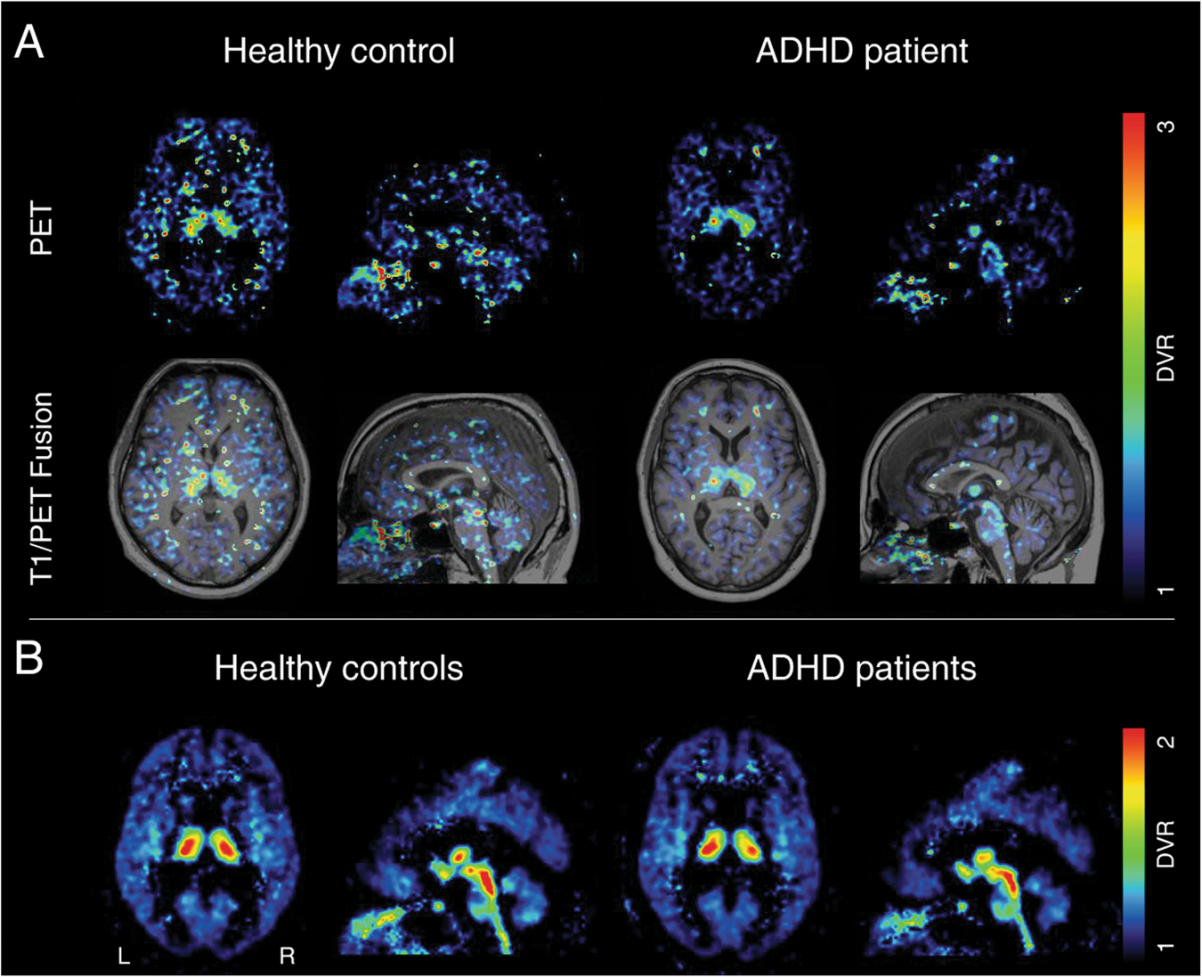
Parametric images of the distribution volume ratio (DVR) in axial and sagittal view after spatial normalization to the MNI-space. **a** An exemplary healthy control and ADHD together with their corresponding T1-MPRAGE image. **b** Group mean of all healthy controls and ADHD patients. ADHD attention-deficit/hyperactivity disorder, MNI Montreal neurological institute, R right, L left.

**Fig. 2:**
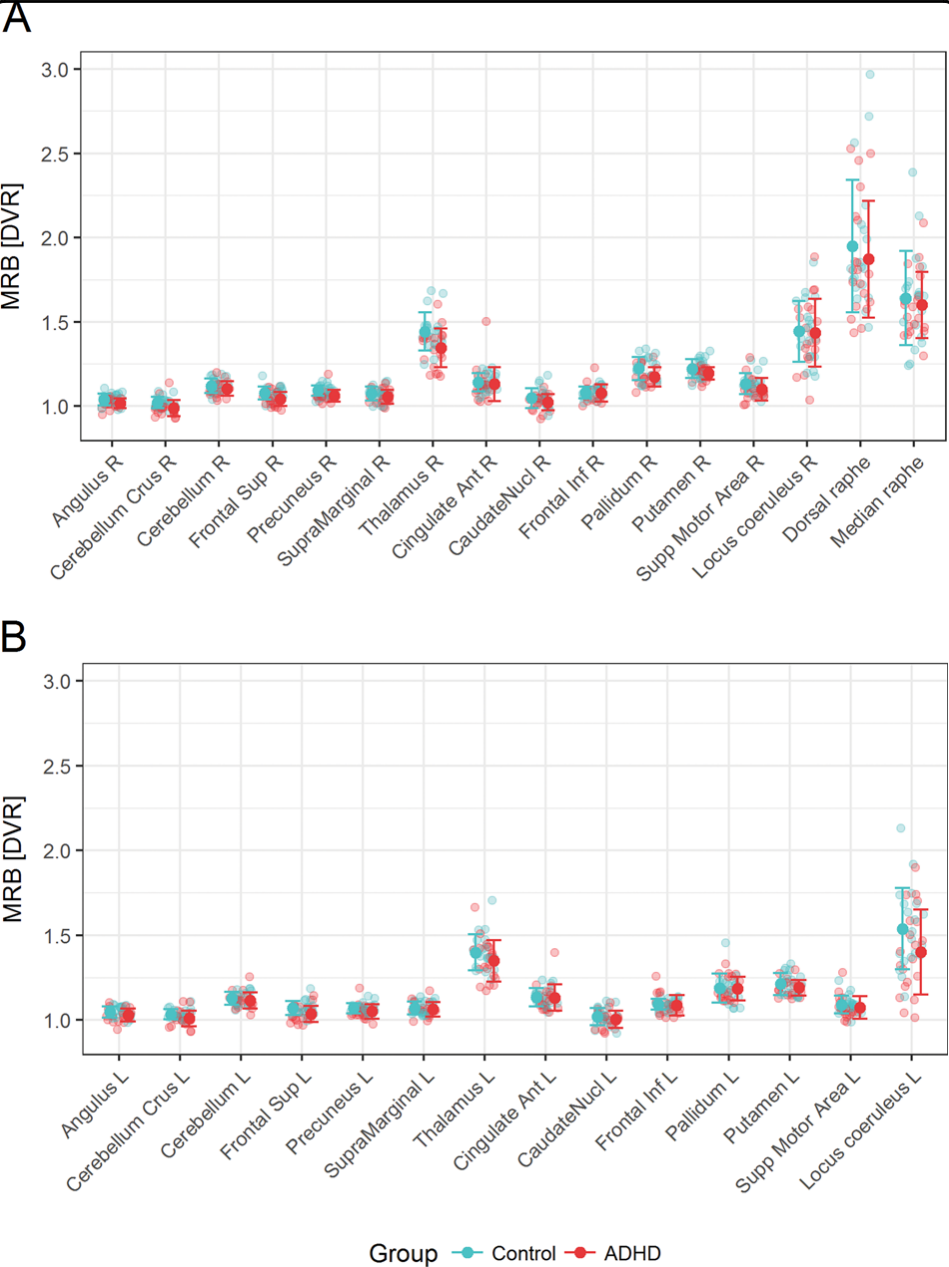
Distribution, mean and error bars of DVR values in selected regions of interest for both, healthy controls (cyan) and ADHD patients (red). **a** Right hemisphere. **b** Left hemisphere. ADHD attention-deficit/hyperactivity disorder, DVR distribution volume ratio, R right, L left.

Examples of parametric images of the DVRs in axial and sagittal view, together with their corresponding T1-MPRAGE images, are presented in Fig. 1. In general, most of the investigated brain regions showed a tendency towards lower values in adult ADHD patients compared with healthy controls. NET-selective MRB density in ADHD and healthy controls was highest in the ascending arousal system (i.e., dorsal raphe > median raphe > LC) and the thalamus (please cf. Figs. 1 and 2).

### VBM analysis

We found no difference in local distribution of gray matter between ADHD patients and control participants.

### NET availability ADHD patients vs. healthy controls

#### Ascending arousal system

MMRM analysis using AAN atlas-based ROIs of the ascending reticular activating system (left, right LC; dorsal, median raphe nuclei) indicated a significant main effect of ROI (*F*_3,38 _= 10.72; *p* < .001) and a significant interaction of group x ROI (*F*_3,38 _= 3.38; *p* = .028) which did not survive adjustment for multiple tests. No main effects were obtained for group (*F*_1,39 _= 0.91; *p* = .347) or BDI (*F*_1,37 _= 0.41; *p* = .525).

#### Attention-related ROIs

In the right fronto-parietal-thalamic-cerebellar ROIs, MMRM analysis revealed a main effect group (*F*_1,25 _= 12.30, *p* = .002), a main effect of ROI (*F*_6,30 _= 143.48; *p* < .001), but no main effect of BDI (*F*_1,21 _= 1.41; *p* = .245) or group × ROI interaction (*F*_6,30 _= 1.74; *p* = .154). When analyzing an analogous model for the left side, a main effect of ROI (*F*_6,38 _= 118.77; *p* < .001) and a main effect group emerged (*F*_1,41 _= 6.80, *p* = .013), but no main effect of BDI (*F*_1,37 _= 1.29; *p* = .264) or group x ROI interaction (*F*_6,38 _= 1.46; *p* = .217).

#### Behavioral control-related ROIs

In the right fronto-basal-ganglia ROIs, MMRM analysis revealed a statistical trend for main effect group (*F*_1,41 _= 3.09, *p* = .086) and group × ROI interaction (*F*_5,38 _= 2.14; *p* = .081), a main effect of ROI (*F*_5,38 _= 138.31; *p* < .001), but no main effect of BDI (*F*_1,37 _= 0.03; *p* = .868). The analogous model for the left side yielded a main effect of ROI (*F*_5,30 _= 104.18; *p* < .001) but no significant main effect group, main effect BDI or group × ROI interaction (.559 ≤ *p* ≤ .706).

### Exploratory correlation analysis

#### Mean DVR in attention-related ROIs and ADHD symptom severity

T-scores of CAARS DSM-IV subscales inattention and hyperactivity/impulsivity are presented in Table 1. Correlation analysis revealed a significant negative association between mean DVR in the right fronto-parietal-thalamic-cerebellar ROIs and ADHD symptom severity (inattention: rho = −0.46; *p* = .003; impulsivity/hyperactivity: rho = −0.40; *p* = .011); participants with lower NET availability scored higher on the CAARS DSM-IV scores, indicating higher symptom severity. The correlation coefficients decreased to −0.01, and 0.10, respectively, when group was included as covariate, which indicated a medium to large size effect of group. Analogous analysis for the left side did not yield any significant findings (inattention: rho = −0.29; *p* = .068; impulsivity/hyperactivity: rho = −0.34; *p* = .034) after adjustment for multiple tests.

#### Mean DVR in attention-related ROIs and neuropsychological measures

**Fig. 3:**
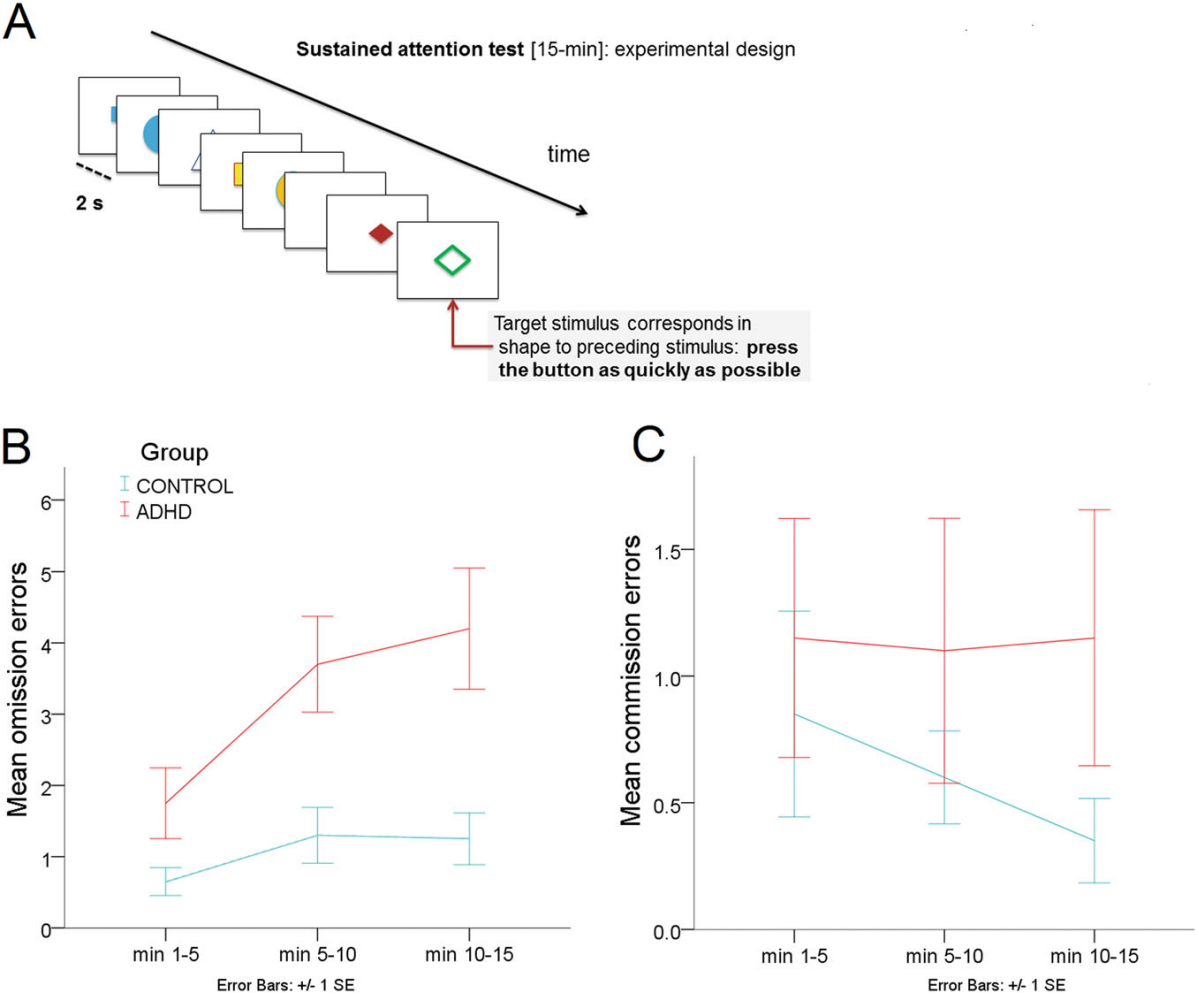
The sustained attention subtest of the computerized test of attentional performance (TAP, Zimmermann and Fimm, 2012). **a** Experimental design. Stimulus onset asynchrony = 2 s. **b** Mean omission errors during the 15-min test in adult ADHD patients (*n* = 20), and age- and sex-matched healthy controls (*n* = 20). **c** Mean commission errors during the 15-min test in adult ADHD patients (*n* = 20) and healthy controls (*n* = 20).

*T*-scores of omission and commission errors during the 15-min sustained attention test were calculated (please cf. Fig. 3). Correlation analysis revealed a significant association between mean DVR in right fronto-parietal-thalamic-cerebellar ROIs and omission errors (rho = 0.43; *p* = .007); participants with lower NET availability had worse performance (=more omission errors) in the sustained attention test.

The correlation coefficient decreased to 0.28 when group was included as covariate, indicating that the effect of group was small. No significant correlations were obtained for mean DVR and commission errors (rho = 0.06; *p* = .732). Analogous analysis for the left side did not survive the correction for multiple tests (omission: rho = 0.35; *p* = .027) and was not significant regarding commission errors (rho = 0.02; *p* = .885).

#### Mean DVR in attention-related ROIs and neurophysiological measures

**Fig. 4:**
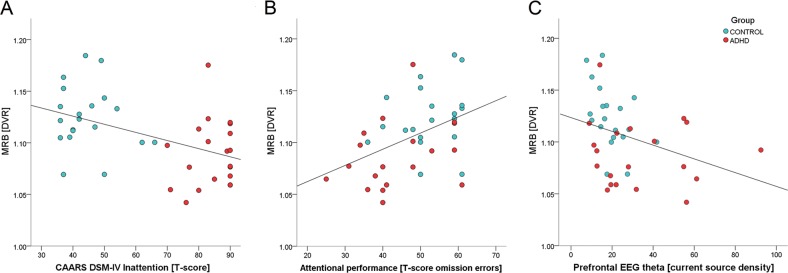
Scatterplots of mean distributed volume ratios (DVR) in right fronto-parietal-thalamic-cerebellar regions of interest versus measures of inattention and EEG extracted theta current density. **a** Inattention assessed with the DSM-IV subscale inattention (total *T*-score) of the Conners’ Adult ADHD Rating Scale Self-Report (CAARS-SR–long version). **b** Inattention assessed with the 15-min subtest of the computerized test of attentional performance (TAP; Zimmermann and Fimm, 2012). *T*-scores of omission errors (number of targets that were presented but not responded to) were used as a measure of sustained attention. **c** Mean absolute right BA9 theta (6.5–8.0 Hz) current density. Red circles: adult ADHD patients (*n* = 20); cyan circles: sex-matched and age-matched healthy controls (*n* = 20). BA Brodmann area.

The current source density values of resting EEG theta in the right and left dorsolateral prefrontal cortices (indexed by current source density of right/left BA9) were calculated. Correlation analysis revealed a significant negative association between mean DVR in right fronto-parietal-thalamic-cerebellar ROIs and theta current density (rho = −0.41; *p* = .009) indicating that participants with lower NET availability had greater theta current density in the right BA9 (please cf. Fig. 4c). The correlation coefficient decreased to −0.32 when group was included as covariate, indicating that the effect of group was small. Analogous analysis for the left side did not survive the correction for multiple tests (rho = −0.31; *p* = .051).

## Discussion

Our main goal was to test the hypothesis that central NET availability is altered in unmedicated adult patients with ADHD compared to age- and sex-matched healthy control subjects. We further explored whether NET availability is associated with ADHD symptom severity or neuropsychological and neurophysiological measures. We obtained several noteworthy findings. In line with our hypothesis, we demonstrated that patients with adult ADHD have decreased NET availability in relevant ROIs for attention, more pronounced in the right hemisphere, in comparison to healthy controls. In addition, in exploratory analyses we showed that lower NET availability in right fronto-parietal-thalamic-cerebellar ROIs is associated with more omission (but not commission) errors in a sustained attention test and greater EEG theta current density in the right dorsolateral prefrontal cortex.

Importantly, we found no differences in local gray matter distribution, which is in line with the systematic review by de Melo et al.^19^. The authors analyzed 17 reviews of whom none reported structural changes in adults with ADHD, while changes were reported in ADHD children. Thus, the obtained differences in NET availability between groups cannot be attributed to structural volume changes in our sample of adult ADHD patients, but rather suggest the involvement of the central LC-NE system corroborating the hypothesis of ADHD as a noradrenergic disorder. This is further supported by the fact that the LC–NE system mediates arousal^11,15^, which is impaired in adult ADHD, as shown in many EEG-studies of brain arousal^16,17,54^. Moreover, drugs, such as methylphenidate and atomoxetine, that modulate the LC-NE system^12^, are effective in ADHD, as demonstrated in randomized controlled clinical trials^55,56^. It is of note that a recent placebo-controlled double-blind fMRI study, testing the comparative neurofunctional effects of methylphenidate and atomoxetine during sustained attention, showed normalization in right fronto-parietal-thalamic areas under methylphenidate, but not under placebo^23^. In line with these findings, in the current study the associations between NET availability in right fronto-parietal-thalamic-cerebellar regions and neuropsychological and neurophysiological parameters, suggest a pathophysiological role of NET availability in adult ADHD and support the model of right hemisphere deficits^19,57^ in ADHD. While brain arousal has been understood as a generalized state of the brain^58^, it is a consistent finding that the ability to maintain an alert state relies heavily on the right cerebral hemisphere^14,59,60^. Our findings of greater right-frontal theta current density in individuals with lower NET availability in attention-related right-hemispheric regions point to a NET-related origin of this deficit. Interestingly, early lesion studies in rats revealed a right hemisphere bias in the LC-NE system^61,62^ indicating a top-down regulation of the NE activation by the right frontal cortex since lesions in this area resulted in lower NE levels in both hemispheres and the LC.

However, functional changes in fronto-basal-ganglia regions on the right side have equally been suggested in ADHD, more often in children^31^, which we could not confirm in the current study of adult ADHD patients. It is conceivable that the fronto-striatal deficits in ADHD children are due to the overall maturation delay^31^ and no longer present in adult ADHD patients. Our findings also do not replicate the findings of Vanicek et al. who found no significant differences in NET binding potential between ADHD and HC using (S,S)-[^18^F]FMeNER-D2 between 22 ADHD patients und 22 HC^33^. Similar to this study, we did not find any differences in NET availability in key regions of the ascending arousal system (i.e., right and left LC). One reason may be that the spatial resolution of the applied method reaches its limitation when quantifying small brain regions such as the LC. As seen in Fig. 2b, the parametric images of the DVRs are able to clearly delineate the hypothalami, while the LC is conflated with the raphe nuclei. In contrast to our approach, the study by Vanicek et al., besides using a different tracer, did not examine any cortical regions, known to be impaired in adult ADHD^20^ and their analyses did not consider the effect of hemisphere. Therefore, the question of consistency cannot be conclusively clarified given the differences in study design.

## Limitations

Some limitations have to be mentioned. The relatively low spatial resolution of PET may have led to a DVR underestimation in the LC. The choice of specific templates (AAL, AAN) may have influenced the results, other parcellation schemes may generate different results, reducing the generalizability of our findings. However, the regional distribution of [^11^C]MRB in the current study is consistent with the known distribution of NET in the brain and in line with previous studies reporting highest distribution in midbrain raphe, the thalamus and the LC^63–65^. Despite these limitations, our results warrant further studies to explore how NE/NET modulates resting-state activity (simultaneously measured by means of echo planar MR sequences) and appropriate behavior as assessed by thorough neuropsychological testing.

## Conclusion

For the first time, the current study demonstrates decreased NET availability in adult ADHD patients in specific brain areas. Our findings indicate the pathophysiological involvement of NET availability in adult ADHD and warrant further clinical intervention studies targeting these specific areas.
